# Effect of Exercise Intensity on Isoform-Specific Expressions of NT-PGC-1 *α* mRNA in Mouse Skeletal Muscle

**DOI:** 10.1155/2014/402175

**Published:** 2014-07-02

**Authors:** Xingyuan Wen, Jing Wu, Ji Suk Chang, Pengcheng Zhang, Jianzhu Wang, Yaliang Zhang, Thomas W. Gettys, Yubin Zhang

**Affiliations:** ^1^State Key Laboratory of Natural Medicines, Department of Biochemistry, China Pharmaceutical University, Nanjing 210009, China; ^2^Laboratory of Nutrient Sensing and Adipocyte Signaling, Pennington Biomedical Research Center, Baton Rouge, LA 70808, USA; ^3^Nanjing University of Chinese Medicine, Nanjing 210023, China

## Abstract

PGC-1*α* is an inducible transcriptional coactivator that regulates mitochondrial biogenesis and cellular energy metabolism in skeletal muscle. Recent studies have identified two additional PGC-1*α* transcripts that are derived from an alternative exon 1 (exon 1b) and induced by exercise. Given that the PGC-1*α* gene also produces NT-PGC-1*α* transcript by alternative 3^′^ splicing between exon 6 and exon 7, we have investigated isoform-specific expression of NT-PGC-1*α* mRNA in mouse skeletal muscle during physical exercise with different intensities. We report here that NT-PGC-1*α*-a mRNA expression derived from a canonical exon 1 (exon 1a) is increased by high-intensity exercise and AMPK activator AICAR in mouse skeletal muscle but not altered by low- and medium-intensity exercise and *β*_2_-adrenergic receptor agonist clenbuterol. In contrast, the alternative exon 1b-driven NT-PGC-1*α*-b (PGC-1*α*4) and NT-PGC-1*α*-c are highly induced by low-, medium-, and high-intensity exercise, AICAR, and clenbuterol. Ectopic expression of NT-PGC-1*α*-a in C_2_C_12_ myotube cells upregulates myosin heavy chain (MHC I, MHC II a) and Glut4, which represent oxidative fibers, and promotes the expression of mitochondrial genes (Cyc1, COX5B, and ATP5B). In line with gene expression data, citrate synthase activity was significantly increased by NT-PGC-1*α*-a in C_2_C_12_ myotube cells. Our results indicate the regulatory role for NT-PGC-1*α*-a in mitochondrial biogenesis and adaptation of skeletal muscle to endurance exercise.

## 1. Introduction

PGC-1*α* is an inducible transcriptional coactivator that regulates cellular energy metabolism and metabolic adaptation to environmental and nutritional stimuli [1–3]. PGC-1*α* is induced in muscle by exercise and regulates energy metabolism and structural adaptation of muscle to exercise [4, 5]. PGC-1*α* stimulates many of the best-known beneficial effects of exercise in muscle: mitochondrial biogenesis, angiogenesis, and fiber-type switching [5, 6]. The health benefits of elevated expression of PGC-1*α* in muscle may go beyond the muscle tissue itself. The contracting muscle secretes myokines that participate in tissue crosstalk. PGC-1*α* promotes the production of newly identified hormone irisin from exercised muscle, increasing energy expenditure with no changes in movement or food intake [7]. Another contraction-induced myokine, beta-aminoisobutyric acid (BAIBA), induces browning of white adipose tissue and increases fat oxidation in the liver [8]. They could be therapeutics for human metabolic disease and other disorders that are improved with exercise.

**Figure 1:**
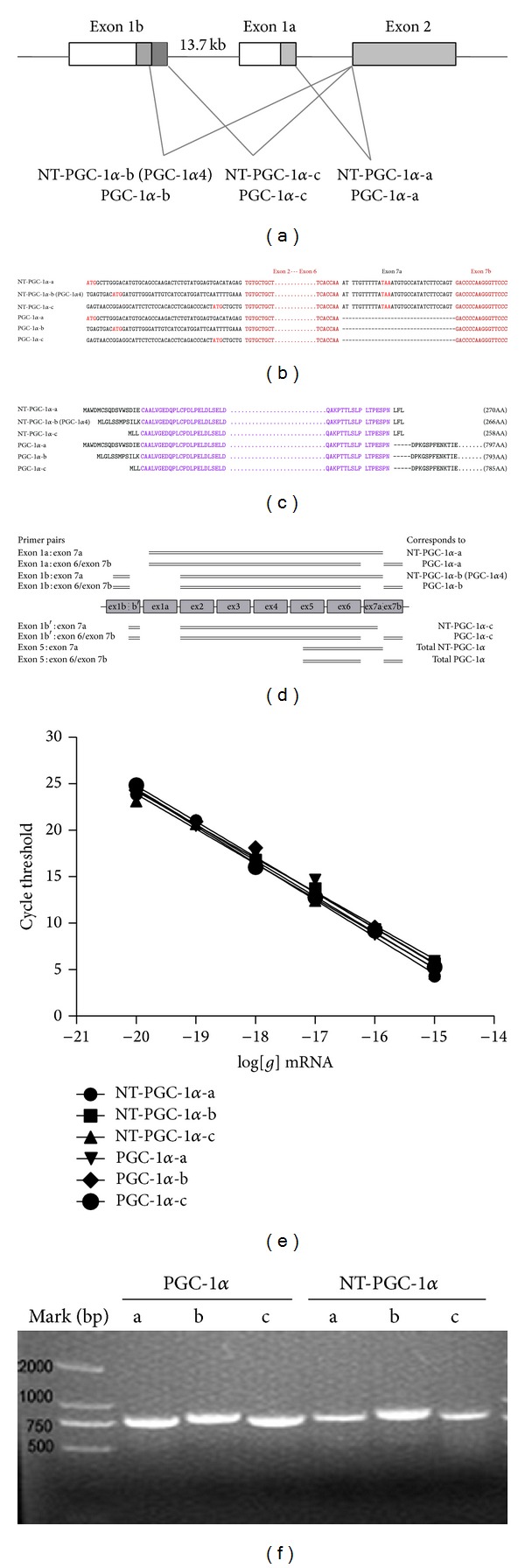
Expression and identification of NT-PGC-1*α*-a, NT-PGC-1*α*-b, and NT-PGC-1*α*-c and PGC-1*α*-a, PGC-1*α*-b, and PGC-1*α*-c isoforms. (a) Schematic structure of the 5′-region of the murine*PGC-1* *α* gene. The schematic structure was slightly adapted from Miura et al. [9]. Boxes indicate promoters, exons, and lines introns. The coding regions and untranslated regions are shown in gray and white, respectively. The proximal promoter (P_Prox._) drives the transcription from exon 1a to produce NT-PGC-1*α*-a mRNA and PGC-1*α*-a mRNA. The alternative promoter (P_Alt._) drives the transcriptions from alternative exon 1b to generate NT-PGC-1*α*-b and PGC-1*α*-c mRNA and PGC-1*α*-b and PGC-1*α*-c mRNA. (b) Three different isoforms of NT-PGC-1*α* (NT-PGC-1*α*-a, NT-PGC-1*α*-b, and NT-PGC-1*α*-c) are produced by different exon usage and alternative 3′ splicing at the intron between exons 6 and 7a which contains an in-frame stop codon TAA (red). Three different isoforms of PGC-1*α* (PGC-1*α*-a, PGC-1*α*-b, and PGC-1*α*-c) are produced by different exon usage and normal 3′ splicing at the intron between exons 6 and 7b. (c) The schematic diagram of amino acid sequences of NT-PGC-1*α*-a, NT-PGC-1*α*-b, and NT-PGC-1*α*-c and PGC-1*α*-a, PGC-1*α*-b, and PGC-1*α*-c. The difference in amino acid sequences among NT-PGC-1*α*-a, NT-PGC-1*α*-b, and NT-PGC-1*α*-c is only in N-terminus shown in gray color, and the remaining sequences of NT-PGC-1*α*-a, NT-PGC-1*α*-b, and NT-PGC-1*α*-c are identical to each other. (d) Schematic diagrams of primer design for detection of NT-PGC-1*α*-a, NT-PGC-1*α*-b, and NT-PGC-1*α*-c mRNA and PGC-1*α*-a, PGC-1*α*-b, and PGC-1*α*-c mRNA. Primer pairs are depicted to the left, exons 1–7 of* PGC-1α* gene are depicted to the middle, and the corresponding names of NT-PGC-1*α* and PGC-1*α* splice variants are stated to the right. Forward primers located in exon 1a (ex 1a) for NT-PGC-1*α*-a and PGC-1*α*-a, exon 1b (ex 1b) for NT-PGC-1*α*-b and PGC-1*α*-b, and exon 1b′-exon 2 (ex 1b′/ex 2) for NT-PGC-1*α*-c and PGC-1*α*-c were combined with reverse primers for NT-PGC-1*α* (ex 7a) and PGC-1*α* (ex 6/ex 7b). Reverse primers for total NT-PGC-1*α* (ex 7a) and total PGC-1*α* (ex 6/ex 7b) were combined with a common forward primer located in the junction of exons 5 and 6 (ex 5/ex 6). The detailed sequences of all primers above mentioned are listed in Table 1. (e) The slopes of the resulting standard curves relating log mass of NT-PGC-1*α*-a, NT-PGC-1*α*-b, NT-PGC-1*α*-c, PGC-1*α*-a, PGC-1*α*-b, and PGC-1*α*-c cDNA to cycle threshold are −3.968, −3.787, −3.748, −3.619, −3.837, and 3.868. (f) RT-PCR products of NT-PGC-1*α*-a, NT-PGC-1*α*-b, and NT-PGC-1*α*-c mRNA and PGC-1*α*-a, PGC-1*α*-b, and PGC-1*α*-c mRNA on agarose gel. The bands correspond to the calculated amplicon sizes: PGC-1*α*-a (770 bp), PGC-1*α*-b (800 bp), PGC-1*α*-c (768 bp), NT-PGC-1*α*-a (796 bp), NT-PGC-1*α*-b (831 bp), and NT-PGC-1*α*-c (799 bp). The normal PCR reaction conditions were 95°C for 10 min, 35 cycles of 95°C for 30 s, 60°C for 30 s, and 72°C for 1 min, and then 72°C for 5 min.

Transcription of the PGC-1*α* gene can be driven by two different promoters in mouse and human skeletal muscle and adipose tissue [9, 10]. A proximal promoter is located upstream of a canonical exon 1 (exon 1a) and a distal promoter followed by an alternative exon 1 (exon 1b) is located ~13.7 kb upstream from the exon 1a of the PGC-1*α* gene (Figure 1(a)) [9]. The PGC-1*α*-b and PGC-1*α*-c transcripts are derived by alternative splicing of the exon 1b at two different 5′ splice sites to the canonical exon 2, producing two PGC-1*α* isoforms, PGC-1*α*-b and PGC-1*α*-c, which actually have different N termini with the first 16 aa of PGC-1*α*-a being replaced by 12 aa and 3 aa alternate protein sequence in PGC-1*α*-b and PGC-1*α*-c, respectively (Figure 1(c)). PGC-1*α*-b and PGC-1*α*-c are functionally active and induce PPAR-dependent transcription to an extent comparable to PGC-1*α*-a. It was also shown that overexpression of PGC-1*α*-b or PGC-1*α*-c in skeletal muscle preferentially upregulated genes involved in mitochondrial biogenesis and fatty acid oxidation [9]. Low-, medium-, and high-intensity exercise and AICAR injection increased PGC-1*α*-b and PGC-1*α*-c transcripts via *β*_2_-AR activation, whereas high-intensity exercise or AICAR injection increased PGC-1*α*-a transcripts independently of *β*_2_-AR activation [11].

Our previous studies have shown that alternative splicing between exons 6 and 7 of the PGC-1*α* gene produces an additional transcript encoding the N-terminal isoform of PGC-1*α* (NT-PGC-1*α*), which consists of 267 amino acids of PGC-1*α* and 3 amino acids from the splicing insert [12]. NT-PGC-1*α* is a functional transcriptional coactivator since it retains the transcription activation and nuclear receptor interaction domains of PGC-1*α* [13–15]. In brown adipose tissue, three NT-PGC-1*α* mRNA isoforms (NT-PGC-1*α*-a, NT-PGC-1*α*-b, and NT-PGC-1*α*-c) derived from exon 1a or exon 1b are coexpressed with three PGC-1*α* isoforms and regulate thermogenic and mitochondrial gene expression in response to cold stress [16].

In this study, we investigated isoform-specific expression of NT-PGC-1*α* mRNA in mouse skeletal muscle in response to different intensities of exercise, AMPK activator AICAR, and *β*_2_-adrenergic receptor agonist clenbuterol. We reported here that NT-PGC-1*α*-a mRNA is induced only by high-intensity exercise and AICAR, whereas NT-PGC-1*α*-b and NT-PGC-1*α*-c mRNAs are induced by low-to-high-intensity exercise, AICAR, and clenbuterol. Moreover, ectopic expression of NT-PGC-1*α*-a activates the muscle transcription program in cultured myotube cells, enhancing mitochondrial oxidative capacity.

## 2. Materials and Methods

### 2.1. Experimental Animals

Six-week-old male C57BL/6J (18–22 g) mice were obtained from SLAC LABORATORY ANIMAL (Shanghai, China). Mice were acclimated for 1 week in a light-controlled room (12 : 12 h light-dark cycle) under a constant temperature (22°C); standard mouse chow and water were available* ad libitum*. All experiments were approved by the Institutional Animal Care and Use Committee at China Pharmaceutical University and adhere to the Jiangsu Provincial Guidelines for the use of experimental animals.

### 2.2. Experimental Protocols

For running exercise experiments, mice were subjected to treadmill running at different intensity exercise, 10 m/min (low-intensity), 20 m/min (medium-intensity), and 30 m/min (high-intensity), for 30 min. Skeletal muscles (gastrocnemius) were isolated at 2 h after exercise [9, 17, 18]. For AICAR experiments, mice were injected with 250 mg/kg bw AICAR (i.p.) or the same volume of saline for control group. Skeletal muscles (gastrocnemius) were isolated at 3 h after AICAR injection [11, 19]. For *β*_2_-agonist experiments, mice were injected subcutaneously with 1 mg/kg bw clenbuterol (Sigma, USA) or the same volume of saline for control group. Skeletal muscles (gastrocnemius) were isolated at 4 h after clenbuterol injection [4, 9, 11]. In all experiments, skeletal muscle (gastrocnemius) was rapidly removed from mice killed by decapitation, frozen immediately in liquid nitrogen, and kept at −80°C until use.

### 2.3. Cell Culture and Transient Transfections

The C_2_C_12_ muscle cells were cultured in Dulbecco's modified Eagle's medium (Gibco, USA) supplemented with 10% fetal bovine serum and penicillin (100 U/mL)/streptomycin (100 *μ*g/mL). When cells reached 80% confluence in six-well plates, they were switched to DMEM containing 2% heat-inactivated horse serum and pen/strep antibiotics for 3 days and differentiated to mature myotube cells. The differentiated cells were treated with AICAR (10^−5^ M) and clenbuterol (10^−5^ M) for 3 h and 1 h, respectively [9, 11, 20]. C_2_C_12_ myotube cells in six-well plate were transiently transfected with 2.0 *μ*g of NT-PGC-1*α*-a/pcDNA3.1 plasmids per well for 24 h using X-tremeGENE HP DNA transfection reagent (Roche, Germany). Then, cells were washed with phosphate-buffered saline and collected and frozen at −80°C until they were assayed.

### 2.4. RNA Isolation and Real-Time PCR (RT-PCR) Assay

Total RNA was extracted from mouse skeletal muscle (gastrocnemius) or C_2_C_12_ cells using TRIzol reagent (Invitrogen, USA). According to the manufacturer's protocol, 50–100 mg of skeletal muscle was mixed with 1 mL of TRIzol and homogenized using Homogenizer (IKA-T-10, Germany). The cells in a well of 6-well plate were mixed with 1 mL of TRIzol. RNA was separated from protein and DNA by the addition of chloroform and precipitated in equal volume of cold pure isopropanol. After a 75% ethanol wash and resuspension in 20 *μ*L of DEPC-treated ddH_2_O, RNA samples were quantified by spectrophotometry. 1 *μ*g of total RNA was reverse-transcribed using Oligo-(dT)_18_ primers and M-MLV reverse transcriptase (Roche, Germany) according to the protocol of First Strand cDNA Synthesis Kit (Roche, Germany) and 50 ng of cDNA was used as template for quantitative RT-PCR on Step One Plus System (Applied Biosystems, USA). The sequences of primers specific for PGC-1*α*-a, PGC-1*α*-b, and PGC-1*α*-c and NT-PGC-1*α*-a, NT-PGC-1*α*-b, and NT-PGC-1*α*-c were designed (Figure 1(d)) and listed in Table 1. RT-PCR reactions were carried out in a 20 *μ*L volume containing 1XFast Start Universal SYBR Green Master (ROX) (Roche, Germany), 50 ng cDNA, 0.3 *μ*M forward and reverse primers (each). qRT-PCR reaction conditions for long products (~800 bp) were 95°C for 10 min and then 40 cycles of 95°C for 15 s, 60°C for 30 s, and 72°C for 1 min [21, 22]. Target gene expression in each sample was normalized to the endogenous control gene cyclophilin. The relative expression among the different conditions (exercise or treatment groups) was determined using the ΔΔ*C*_*T*_ method as outlined in the Applied Biosystems protocol for RT-PCR.

### 2.5. Western Blot Analysis

C_2_C_12_ cells were homogenized and sonicated in ice-cold lysis buffer (RIPA) containing 50 mM Tris/HCl (pH 7.4), 150 mM NaCl, 1% SDS, 0.1% Triton X-100, 0.5% sodium deoxycholate, and protease inhibitor cocktail (Roche, Switzerland). Cell lysates were centrifuged at 12,000 ×g, supernatant was collected, and protein concentration was determined with bicinchoninic acid (BCA) method. Equal amounts of total protein (100 *μ*g) were loaded on 10% polyacrylamide gel (29 : 1 acrylamide-bisacrylamide), separated by SDS-PAGE, and transferred to PVDF membranes (Millipore, USA). The membrane was blocked for 1 h at room temperature in TBST buffer (50 mM Tris/HCl, 150 mM NaCl (pH 7.4), and 0.1% Tween-20) containing 5% nonfat dried milk. The membrane was incubated with the appropriate primary antibody in TBST with 5% nonfat dried milk overnight at 4°C. Primary antibody anti-PGC-1*α* mouse mAb (ST1202) (Calbiochem, USA) andanti-PGC-1*α* rabbit polyclonal antibody (SC-13067) (Santa Cruz, USA) were used to detect total NT-PGC-1*α* due to the commercial antibody against the same N-terminal sequence of PGC-1*α* and NT-PGC-1*α*. The antibody can only detect total PGC-1*α* and NT-PGC-1*α* but cannot distinguish each isoform of PGC-1*α* and NT-PGC-1*α*. Immunoblots were hybridized with antibody raised against *β*-tubulin (Santa Cruz, USA) as loading control. The membrane was then washed 3 times with TBST and incubated for 1 h with HRP-conjugated secondary antibody. Finally, the immunoreactive proteins were visualized using chemiluminescence reagent (Amersham Biosciences, USA).

### 2.6. Citrate Synthase (CS) Assay

Citrate synthase activity was determined using a commercially available kit (Genmed, China). The principle of the assay is the reaction of acetyl-CoA with OAA (oxaloacetate) and the release of free CoA-SH, which reacts with a colorimetric reagent, DTNB [5,5-dithiobis(2-nitrobenzoate)], to form yellow colored compound (5-thio-2-nitrobenzoic acid (TNB)) [23]. The procedures were applied based on the manufacture's guideline. Briefly, C_2_C_12_ myotube cells transfected with NT-PGC-1*α*-a/pcDNA3.1 were collected and lysed to disrupt the mitochondria and expose the CS. The supernatants were collected by centrifugation at 16,000 g for 5 min at 4°C. The protein concentration was measured by BCA method. 10 *μ*L (20 *μ*g) of total protein containing CS was added to test well containing reaction substrates reagent and 10 *μ*L of negative control reagent was added to control well. The formation of 5-thio-2-nitrobenzoic acid was measured spectrophotometrically at 412 nm in 96-well format by using a microplate reader (Bio-TeK, USA) at 0 min and 15 min. All measurements were performed in duplicate in the same setting at 20–22°C. The CS activity was then normalized to the total protein content and was reported in as nanomoles per milligram protein per minute.

### 2.7. Statistical Analysis

Values are expressed as means ± S.E. Statistically significant differences were determined using unpaired Student's *t*-tests. Statistical differences were considered significant if *P* < 0.05.

## 3. Results

### 3.1. Expression and Identification of NT-PGC-1*α* and PGC-1*α* Isoforms in Mouse Skeletal Muscle

Three PGC-1*α* isoforms, PGC-1*α*-a, PGC-1*α*-b, and PGC-1*α*-c, which are derived from either exon 1a or exon 1b, have been reported in skeletal muscle [9, 10]. In addition, the expressions of NT-PGC-1*α*-a, NT-PGC-1*α*-b, and NT-PGC-1*α*-c isoforms have been reported in brown adipose tissue [16]. They all arise from different promoter usage and alternative internal splicing (Figures 1(a), 1(b), and 1(c)). Thus, these previous studies suggest that the single PGC-1*α* gene can produce six different transcripts in skeletal muscle. To explore the isoform-specific expression of NT-PGC-1*α* mRNA in skeletal muscle, specific primers (Table 1) were designed to detect only NT-PGC-1*α*-a, NT-PGC-1*α*-b, and NT-PGC-1*α*-c mRNA as described in Figure 1(d). In addition, we designed a set of primers that detect only PGC-1*α*-a, PGC-1*α*-b, and PGC-1*α*-c mRNA (Figure 1(d)). The expression levels of NT-PGC-1*α*-a, NT-PGC-1*α*-b, and NT-PGC-1*α*-c and PGC-1*α*-a, PGC-1*α*-b, and PGC-1*α*-c can be quantitatively determined by qRT-PCR. The slopes of the resulting standard curves relating log mass of NT-PGC-1*α*-a, NT-PGC-1*α*-b, NT-PGC-1*α*-c, PGC-1*α*-a, PGC-1*α*-b, and PGC-1*α*-c cDNA to cycle threshold are −3.968, −3.787, −3.748, −3.619, −3.837, and 3.868 (Figure 1(e)). Each PCR product amplified by a specific set of primers was checked by agarose gel analysis (Figure 1(f)) and the specificity of PCR products was confirmed by DNA sequencing (data not shown).

### 3.2. Exercise Intensity-Dependent Expression of NT-PGC-1*α* mRNA Isoforms in Skeletal Muscle

**Figure 2:**
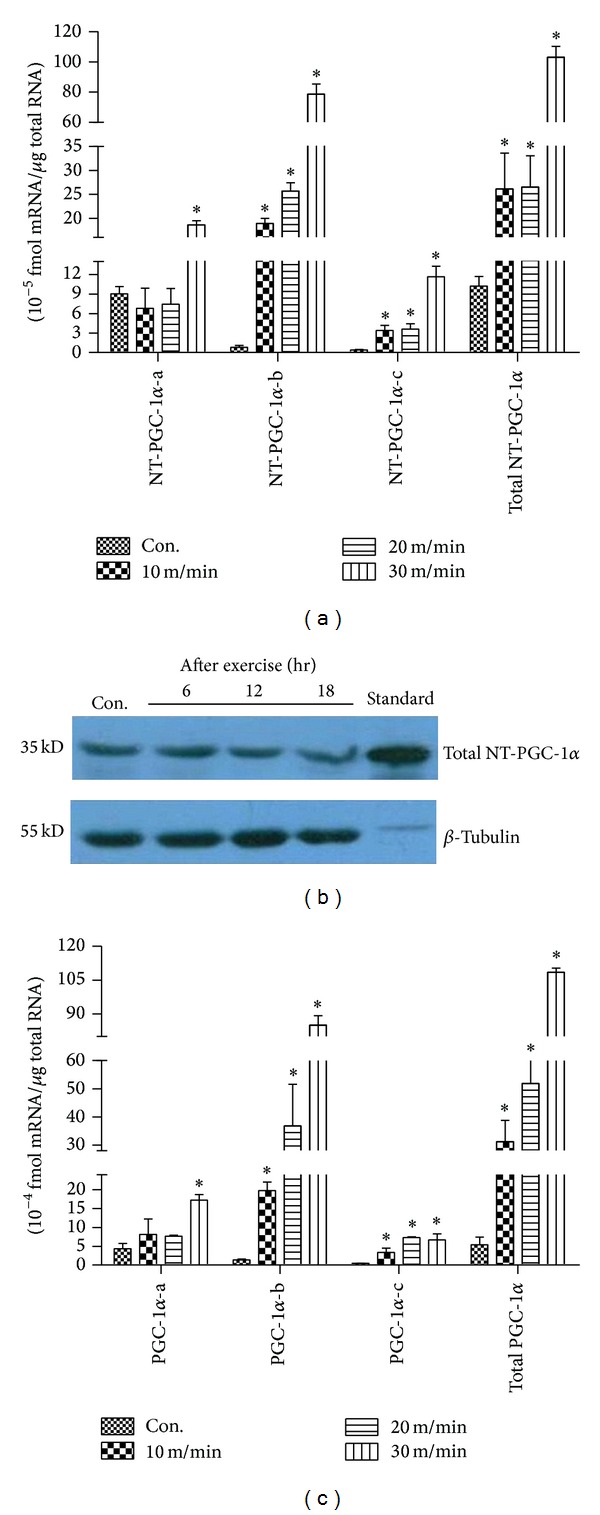
Isoform-specific expression of NT-PGC-1*α*-a, NT-PGC-1*α*-b, and NT-PGC-1*α*-c and PGC-1*α*-a, PGC-1*α*-b, and PGC-1*α*-c mRNA in response to different intensities of exercise. (a) Induction of NT-PGC-1*α*-a, NT-PGC-1*α*-b, and NT-PGC-1*α*-c mRNA and total NT-PGC-1*α* mRNA in skeletal muscle (gastrocnemius) at low-, medium-, and high-intensity exercise in C57BL/6J mice. (b) Induction of total NT-PGC-1*α* protein expression in skeletal muscle (gastrocnemius) in C57BL/6J mice by exercise. C57BL/6J mice were subjected to treadmill running exercise at 20 m/min for 30 min. Gastrocnemius were separated 6 h, 12 h, and 18 h after exercise. Protein expression was analyzed by Western blotting, and *β*-tubulin was blotted as a loading control. (c) Induction of PGC-1*α*-a, PGC-1*α*-b, and PGC-1*α*-c mRNA and total PGC-1*α* mRNA in the same tissue and conditions above. Control mice were kept in cage without food. Gastrocnemius were separated 2 h after exercise. Relative gene expression was quantified by real-time PCR and relative to amount of each cDNA, compared to control mice. Values are means ± S.E. (*n* = 8). *P < 0.05 versus ctrl. Data are representative of three experiments.

To examine whether the expression of NT-PGC-1*α* mRNA isoforms is differentially induced in skeletal muscle in response to different intensities of exercise, C57BL/6J mice were subjected to treadmill running exercise at different intensities, 10 m/min (low-intensity), 20 m/min (medium-intensity), and 30 m/min (high-intensity), for 30 min according to the exercise program. Basal mRNA levels of NT-PGC-1*α*-b and NT-PGC-1*α*-c were very low when compared with that of NT-PGC-1*α*-a in control mice (Figure 2(a)). At low- and medium-intensity exercise, expression of NT-PGC-1*α*-a mRNA was not altered, but expression of NT-PGC-1*α*-b and NT-PGC-1*α*-c mRNA was significantly elevated (Figure 2(a)). After high-intensity exercise, NT-PGC-1*α*-b and NT-PGC-1*α*-c mRNA showed 97-fold and 28-fold increases in expression, respectively, whereas NT-PGC-1*α*-a mRNA levels increased by 2.1-fold (Figure 2(a)). It has been reported that a single exercise bout resulted in an increment in PGC-1 protein concentration [24]. In this study, we found that total NT-PGC-1*α* protein is a constitutive expression in skeletal muscle and is also increased by exercise (Figure 2(b)). In parallel, PGC-1*α* mRNA isoforms had a similar isoform-specific expression pattern in response to different intensities of exercise (Figure 2(c)). Taken together, these data illustrate that three NT-PGC-1*α* transcripts are coexpressed with PGC-1*α* isoforms in mouse skeletal muscle, but their relative expression levels are dependent on exercise intensity.

### 3.3. AMPK Activator AICAR Increases the Expression of Three NT-PGC-1*α* mRNAs in Skeletal Muscle

**Figure 3:**
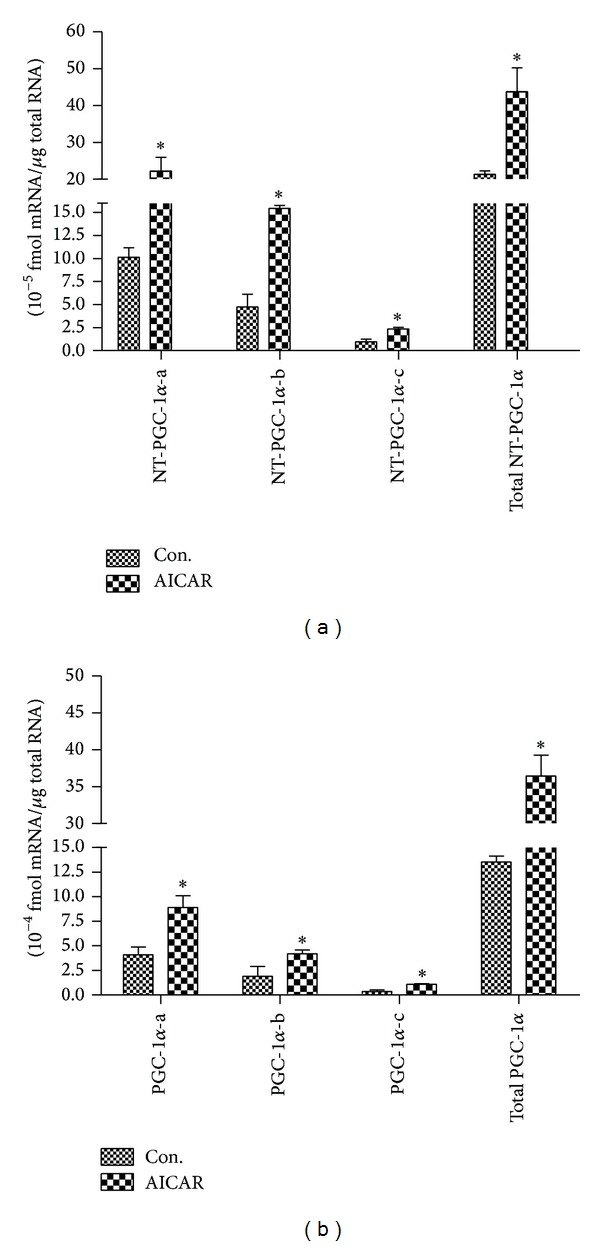
The effect of AMPK activation on NT-PGC-1*α*-a, NT-PGC-1*α*-b, and NT-PGC-1*α*-c and PGC-1*α*-a, PGC-1*α*-b, and PGC-1*α*-c mRNA expression in mouse skeletal muscle. (a) Induction of NT-PGC-1*α*-a, NT-PGC-1*α*-b, and NT-PGC-1*α*-c mRNA and total NT-PGC-1*α* mRNA in skeletal muscle (gastrocnemius) of C57BL/6J mice by the subcutaneous injection of AMPK agonist AICAR at a dose of 250 mg/kg bw. (b) Induction of PGC-1*α*-a, PGC-1*α*-b, and PGC-1*α*-c mRNA and total PGC-1*α* mRNA in the same tissue and conditions above. Control mice were injected by saline. Gastrocnemius were separated 3 h after injection. Relative gene expression was quantified by real-time PCR and relative to amount of each cDNA, compared to control mice. Values are means ± S.E. (*n* = 6). *P < 0.05 versus ctrl. Data are representative of three experiments.

AICAR is a pharmacological activator of AMPK showing similar effects of exercise for increasing PGC-1*α* expression [11, 19], mitochondrial biogenesis, and fatty acid oxidation in skeletal muscle. To examine the effect of AMPK activation on isoform-specific expression of NT-PGC-1*α* mRNA in skeletal muscle, mice were injected i.p. with 250 mg/kg body weight AICAR or the same volume of saline. Skeletal muscles (gastrocnemius) were isolated after AICAR injection for 3 h, and gene expression was analyzed by qPCR. The expressions of NT-PGC-1*α*-a, NT-PGC-1*α*-b, and NT-PGC-1*α*-c mRNA were increased by 2.2-fold, 3.2-fold, and 2.4-fold, respectively (Figure 3(a)). In addition, a similar increase in PGC-1*α* isoforms expression was observed (Figure 3(b)). These data illustrate that the proximal and alternative promoters are similarly activated by AMPK activation to produce exon 1a- and 1b-derived NT-PGC-1*α* and PGC-1*α* transcripts.

### 3.4. *β*_2_-AR Agonist Clenbuterol Increases the Expression of NT-PGC-1*α*-b and NT-PGC-1*α*-c but Not NT-PGC-1*α*-a in Skeletal Muscle

**Figure 4:**
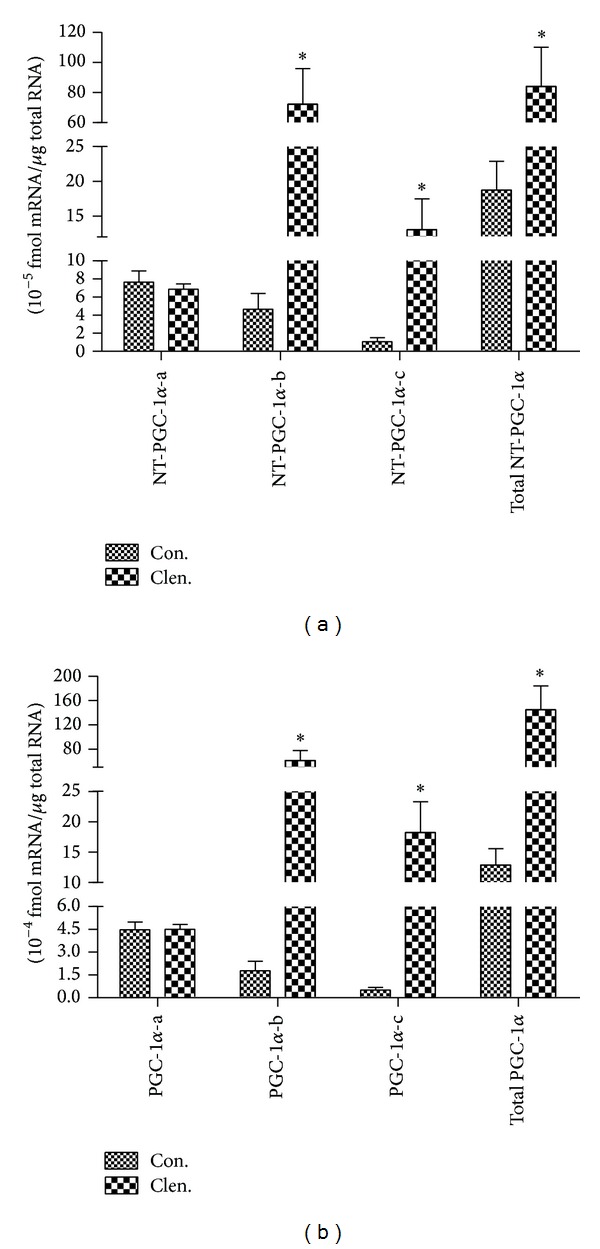
The effect of *β*_2_-AR activation on NT-PGC-1*α*-a, NT-PGC-1*α*-b, and NT-PGC-1*α*-c and PGC-1*α*-a, PGC-1*α*-b, and PGC-1*α*-c mRNA expression. (a) Expression of NT-PGC-1*α*-a, NT-PGC-1*α*-b, and NT-PGC-1*α*-c mRNA and total NT-PGC-1*α* mRNA in skeletal muscle (gastrocnemius) of C57BL/6J mice by the subcutaneous injection of *β*_2_-AR agonist clenbuterol at a dose of 1 mg/kg bw. (b) Expression of PGC-1*α*-a, PGC-1*α*-b, and PGC-1*α*-c mRNA and total PGC-1*α* mRNA in the same tissue and conditions above. Control mice were injected by saline. Gastrocnemius were separated 4 h after injection. Relative gene expression was quantified by real-time PCR and relative to amount of each cDNA, compared to control mice. Values are means ± S.E. (*n* = 6). *P < 0.05 versus ctrl. Data are representative of three experiments.

Given that *β*_2_-adrenergic receptor agonist clenbuterol increases PGC-1*α* expression in skeletal muscle [4, 9, 11], we examined the effect of clenbuterol on isoform-specific expression of NT-PGC-1*α* mRNA. *β*_2_-AR agonist clenbuterol was subcutaneously injected to C57BL/6J mice at a dose of 1 mg/kg body weight in our experiments. The expressions of NT-PGC-1*α*-b and NT-PGC-1*α*-c mRNA were significantly increased by clenbuterol, whereas there was no change in mRNA levels of NT-PGC-1*α*-a (Figure 4(a)). Similarly, PGC-1*α*-b and PGC-1*α*-c mRNAs, but not PGC-1*α*-a mRNA, were largely induced by clenbuterol in skeletal muscle (Figure 4(b)), indicating that the alternative promoter of the PGC-1*α* gene is more responsive to *β*_2_-AR signaling.

### 3.5. NT-PGC-1*α*-a Enhances Mitochondrial Oxidative Function in C_2_C_12_ Myotube Cells

**Figure 5:**
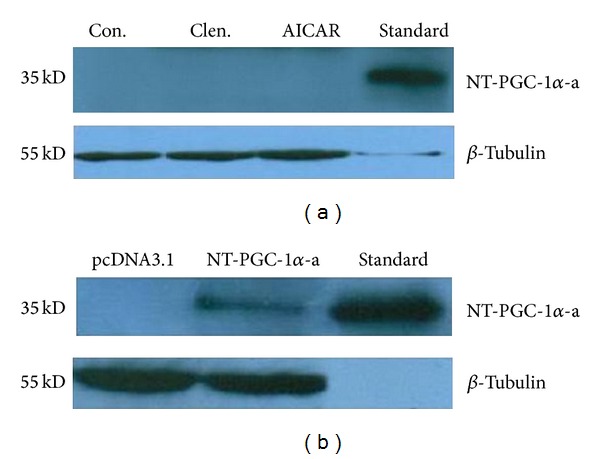
Ectopic expression of NT-PGC-1*α*-a protein in C_2_C_12_ myotubes. (a) No expression of endogenous NT-PGC-1*α* protein in differentiated C_2_C_12_ myotube cells induced by 10^−5^ M AICAR for 3 h and 10^−5^ M clenbuterol for 1 h. Control cells were treated with vehicle. (b) Expression of exogenous NT-PGC-1*α*-a protein in C_2_C_12_ myotube cells by transfection with plasmid NT-PGC-1*α*-a/pcDNA3.1. Control cells were transfected with pcDNA3.1 empty vector. Western blot analysis of NT-PGC-1*α*-a using an antibody raised against the N-terminal region of PGC-1*α* (aa 1–300). *β*-Tubulin was blotted as a loading control.

A recent study reported a muscle-specific PGC-1*α* isoform, PGC-1*α*4, which has shown to be induced by resistance training [17]. PGC-1*α*4 is actually identical to NT-PGC-1*α*-b, which is derived from exon 1b of the PGC-1*α* gene. NT-PGC-1*α*-b (PGC-1*α*4) has shown to induce muscle cell hypertrophy but not mitochondrial biogenesis [17]. Given that NT-PGC-1*α*-a is induced by exercise in mouse skeletal muscle, we explored whether NT-PGC-1*α*-a regulates the transcription program in skeletal muscle. To test NT-PGC-1*α*-a function, differentiated C_2_C_12_ cells were used as a myotube cell model since no detectable NT-PGC-1*α*-a mRNA (data not shown) and protein (Figure 5(a)) were observed in differentiated C_2_C_12_ cells even after treatment with either AICAR or clenbuterol. Thus, overexpression of exogenous NT-PGC-1*α*-a was carried out by transfection of differentiated C_2_C_12_ cells with NT-PGC-1*α*-a (Figure 5(b)).

**Figure 6:**
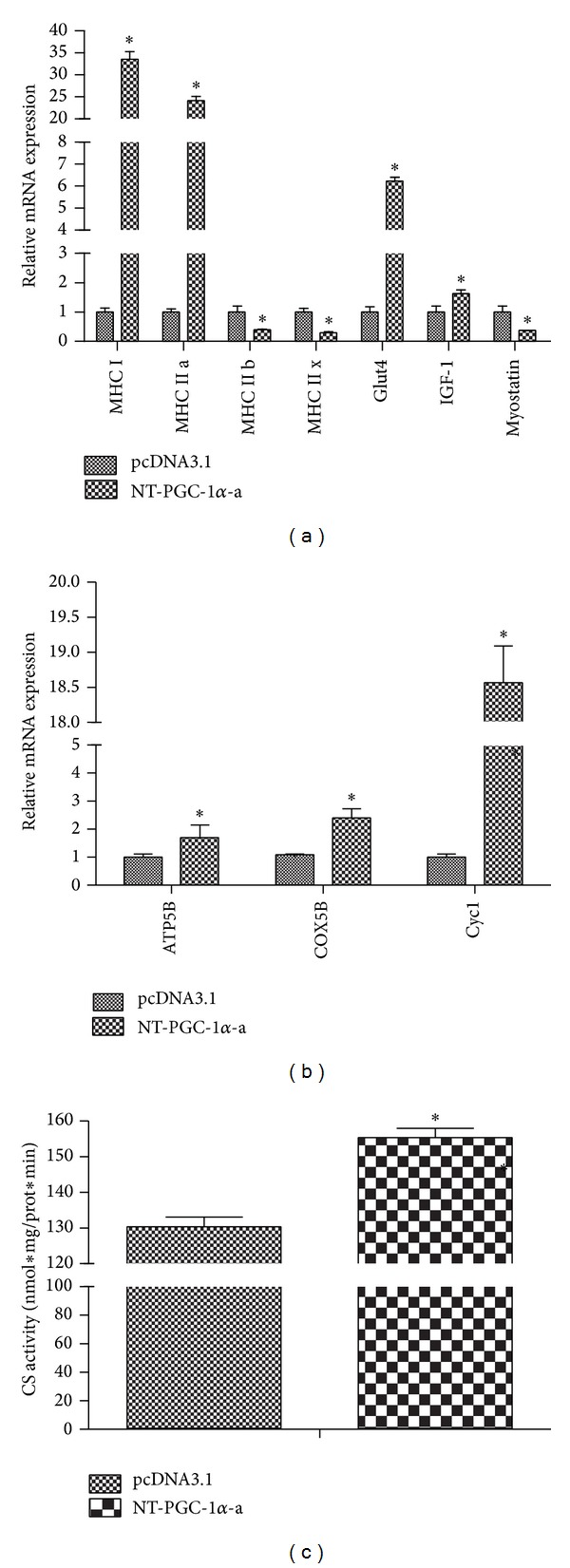
*In vitro *functional analysis of NT-PGC-1*α*-a. (a) NT-PGC-1*α* promotes oxidative phenotyping myotube fiber and the skeletal muscle mass growth. NT-PGC-1*α*-a induces the expression of MHC I, MHC II a, Glut4, and IGF-1 mRNA and inhibits the expression of MHC II b, MHC II x, and Myostatin mRNA in differentiated C_2_C_12_ cells transfected with plasmid NT-PGC-1*α*-a/pcDNA3.1. (b) NT-PGC-1*α*-a increases mitochondrial function through induction of ATP5B, COX5B, and Cyc1 mRNA expression in the same cells and conditions above. Relative gene expression was quantified by real-time PCR and normalized to the expression of cyclophilin with *C*_*T*_ method, compared to control cells transfected with empty vector pcDNA3.1. Values are means ± S.E. (*n* = 3). *P < 0.05 versus ctrl. (c) NT-PGC-1*α*-a enhances the activity of citrate synthase in C_2_C_12_ myotube cells transfected with plasmid NT-PGC-1*α*-a/pcDNA3.1. The activity is normalized to the total protein content of the sample used in the assay. Normalized data are presented as means ± S.E. *P < 0.05 versus ctrl. (*P < 0.05, significantly different from control).

There are four muscle fiber types, Type I, Type II a, Type II b, and Type II x, which can be distinguished by the type of myosin heavy chain (MHC) isoform(s) [25, 26]. Type I fibers are referred to as being “slow twitch oxidative.” Type II a fibers are “fast twitch oxidative.” Type II b fibers are “fast twitch glycolytic,” and Type II x fibers are intermediate to Type II a and II b fibers. Type I fibers have an oxidative profile with high mitochondrial content, capillary density, and glucose transporter 4 (Glut4) expression sensitive to insulin. We found that NT-PGC-1*α*-a promoted the expression of MHC I, MHC II a, and Glut4 in differentiated C_2_C_12_ cells by more than 30-fold, 20-fold, and 6-fold, respectively (Figure 6(a)), but decreased the expression of MHC II b and MHC II x by 60 percent and 70 percent (Figure 6(a)). These results indicate that NT-PGC-1*α*-a has a potential role in skeletal muscle adaptation to a more oxidative phenotype during endurance exercise.

IGF-1 plays an essential role in muscle development, muscle cell proliferation, and muscle growth [27]. Myostatin is a negative regulator of muscle growth and functions by inhibiting myoblast proliferation [28]. We found that NT-PGC-1*α*-a promoted the expression of IGF-1 in differentiated C_2_C_12_ cells by more than 1.5-fold (Figure 6(a)) but decreased the expression of myostatin by 70 percent (Figure 6(a)). These data demonstrated that NT-PGC-1*α*-a has a potential role in muscle development, muscle cell proliferation, and muscle growth.

To assess whether NT-PGC-1*α*-a expression alters mitochondrial biogenesis and function, several markers of mitochondrial biogenesis and function were examined in NT-PGC-1*α*-a-transfected C_2_C_12_ myotube cells. The mRNA expressions of mitochondrial component genes, Cyc1, COX5B, and ATP5B, were upregulated by 18.9-, 2-, and 1.5-fold, respectively, when compared with control C_2_C_12_ cells transfected by an empty vector pcDNA3.1 (*P* < 0.05; Figure 6(b)). These nuclear DNA-encoded mitochondrial proteins are critical for mitochondrial biogenesis and oxidative metabolism. Citrate synthase is one of the key regulatory enzymes in an energy-generating metabolic pathway [23]. It has been extensively used as a metabolic marker for assessing mitochondrial oxidative capacity. The activity of citrate synthase in NT-PGC-1*α*-transfected C_2_C_12_ myotube cells was 1.4-fold higher than that of control C_2_C_12_ cells (Figure 6(c)). This is consistent with the studies reporting the acute increase in CS activity in skeletal muscle after a single bout of exercise [23].

## 4. Discussion

The PGC-1*α* gene has been thought to produce single transcript and protein. However, recent studies reported that multiple mRNAs are produced from the PGC-1*α* gene by different exon usage and alternative splicing in skeletal muscle and brown adipose tissue [9, 10]. We show here that NT-PGC-1*α*-a, NT-PGC-1*α*-b, and NT-PGC-1*α*-c are coexpressed with PGC-1*α*-a, PGC-1*α*-b, and PGC-1*α*-c in mouse skeletal muscle. Consistent with the previous findings on PGC-1*α* expression in mouse skeletal muscle, isoform-specific expression of NT-PGC-1*α* mRNA was dependent on exercise intensity [11]. Exon 1a-driven NT-PGC-1*α*-a was induced by both high-intensity exercise and AMPK activation but not by *β*_2_-AR stimulation, whereas expression of exon 1b-driven NT-PGC-1*α*-b and NT-PGC-1*α*-c were markedly elevated by low-to-high-intensity exercise, AICAR, and clenbuterol. Transcriptional shift between exon 1a and exon 1b of the PGC-1*α* gene may imply isoform-specific roles for NT-PGC-1*α* in basal and exercised conditions. The further studies on the underlying mechanism involving transcription factors on the proximal and alternative promoters and the physiological regulation of proximal and alternative promoters are warranted. In addition, it is also important to explore the molecular mechanism and regulation of the alternative splicing that produce NT-PGC-1*α* from posttranscription modification of PGC-1*α* pre-mRNA in different physiological and pathological conditions.

The exercise-inducible and exon 1b-driven NT-PGC-1*α*-b is actually identical to a recently identified muscle-specific PGC-1*α* isoform, PGC-1*α*4 [17]. PGC-1*α*4 has shown to be induced by resistance training, but not by endurance training, and regulate skeletal muscle mass [17]. However, we observed here that NT-PGC-1*α*-b expression is largely elevated by low-to-high endurance exercise, AMPK activation, and *β*_2_-AR activation in mouse skeletal muscle. In agreement with our findings, a recent study has reported that NT-PGC-1*α*-b (PGC-1*α*4) is induced by both endurance and resistance exercise in human skeletal muscle [18]. Moreover, PGC-1*α*-b expression was largely elevated by endurance exercise, AMPK activation, and *β*_2_-AR activation in mouse skeletal muscle. Skeletal muscle-specific expression of PGC-1*α*-b has shown to increase mitochondrial biogenesis and exercise capacity [9, 29].

Type I fibers containing MHC I are referred to as being “slow twitch oxidative” and have an oxidative profile with high mitochondrial content, capillary density, and glucose transporter 4 (Glut4) expression sensitive to insulin. Type II a fibers expressing MHC II a are fatigue resistant and have the highest oxidative (aerobic) capacity of the fast fibers with numerous mitochondria. Type II b fibers are the fastest contracting and fatiguing fibers which contain Type MHC II b and few mitochondria, using primarily glycolytic (anaerobic) pathways to generate energy for contraction [25, 26]. Type I fibers are characterized by low force/power/speed production and high endurance, Type II b and Type II x fibers are characterized by high force/power/speed production and low endurance, while Type II a fibers fall in between [26]. Here, we clearly showed that overexpression of NT-PGC-1*α*-a in cultured C_2_C_12_ myotubes upregulates the expression of myosin heavy chain I and II a (MHC I, MHC II a), glucose transporter 4 (Glut4), and insulin-like growth factor 1 (IGF-1), while reducing MHC II b, MHC II x, and myostatin mRNA expression. Furthermore, NT-PGC-1*α*-a induced the expression of mitochondrial respiratory subunit (COX5B, Cyc1, and ATP5B) and increased the mitochondrial oxidative enzyme CS activities. However, NT-PGC-1*α*-b (PGC-1*α*4) and PGC-1*β* have shown to induce the expression of MHC II x, but not MHC I, in skeletal muscle [17, 30]. Furthermore, NT-PGC-1*α*-b (PGC-1*α*4) has shown to promote muscle development, muscle cell proliferation, and muscle growth [17]. These data suggested that NT-PGC-1*α*-a promotes muscle fiber switching to more oxidative phenotype in gastrocnemius with increased mitochondrial biogenesis and muscle growth. The presence and differential expression of multiple PGC-1*α* and NT PGC-1*α* isoforms may enhance the ability of skeletal muscle to optimally adapt to different intensities of exercise.

## Figures and Tables

**Table 1:** Primer sequences for PCR analysis.

Gene	Forward primer	Reverse primer
Total PGC-1*α*	5′-TGC CAT TGT TAA GAC CGA G-3′	5′-GGT CAT TTG GTG ACT CTG G-3′
Total NT-PGC-1*α*	5′-TGC CAT TGT TAA GAC CGA G-3′	5′-GGT CAC TGG AAG ATA TGG C-3′
NT-PGC-1*α*-a	5′-TTG ACT GGC GTC ATT CG-3′	5′-CTG GAA GAT ATG GCA CAT-3′
NT-PGC-1*α*-b	5′-CAT GGA TTC AAT TTT GAA ATG-3′	5′-GGT CAC TGG AAG ATA TGG C-3′
NT-PGC-1*α*-c	5′-CTA TGC TGC TGT GTG CTG-3′	5′-GGT CAC TGG AAG ATA TGG C-3′
PGC-1*α*-a	5′-TTG ACT GGC GTC ATT CG-3′	5′-GGT CAT TTG GTG ACT CTG G-3′
PGC-1*α*-b	5′-CAT GGA TTC AAT TTT GAA ATG-3′	5′-GGT CAT TTG GTG ACT CTG G-3′
PGC-1*α*-c	5′-CTA TGC TGC TGT GTG CTG-3′	5′-GGT CAT TTG GTG ACT CTG G-3′
Glut4	5′-GTT GGT CTC GGT GCT CTT AGT-3′	5′-ATA GCA TCC GCA ACA TAC TGG-3′
ATP5B	5′-GGC ACT GAA GGC TTG GTT AG-3′	5′-CAA GAG AAG ATT CTC AGC GAC-3′
Cyc1	5′-CCC TGA CCT CAG CTA CAT C-3′	5′-CAA GAG AAG ATT CTC AGC GAC-3′
COX5B	5′-GCT GCA TCT GTG AAG AGG ACA AC-3′	5′-CAG CTT GTA ATG GGT TCC ACA GT-3′
MHC I	5′-CCA AGG GCC TGA ATG AGG AG-3′	5′-GCA AAG GCT CCA GGT CTG AG-3′
MHC II a	5′-AAG CGA AGA GTA AGG CTG TC-3′	5′-GTG ATT GCT TGC AAA GGA AC-3′
MHC II b	5′-CGA AGG CGG AGC TAC GGT CA-3′	5′-CGG CAG CCA CTT GTA GGG GT-3′
MHC II x	5′-GCC AGG GTC CGT GAA CTT GAA G-3′	5′-CCT CCG CTT CCT CAG CTT GTC T-3′
IGF-1	5′-AAA GCA GCC CGC TCT ATC C-3′	5′-CTT CTG AGT CTT GGG CAT GTC A-3′
Myostatin	5′-GGC CAT GAT CTT GCT GTA AC-3′	5′-TTG GGT GCG ATA ATC CAG TC-3′
Cyclophilin	5′-CCA TCG TGT CAT CAA GGA CTT CAT-3′	5′-CTT GCC ATC CAG CCA GGA GGT CTT-3′

PGC-1*α*: peroxisome proliferators-activated receptor-*γ* coactivator-1*α*; NT-PGC-1*α*: N-truncated PGC-1*α*; Glut4: glucose transporter 4; ATP5B: ATP synthase B subunit; Cyc1: cytochrome C unit 1; COX5B: cytochrome oxidase 5 B subunit; MHC I: myosin heavy chain I; MHC II a: myosin heavy chain II a; MHC II b: myosin heavy chain II b; MHC II x: myosin heavy chain II x; Cyclophilin B: peptidylprolyl isomerase B; IFG-1: insulin-like growth factor 1.
